# The dynamics of coordination patterns in multiteam systems response to emergency: Case study from a pharmaceutical enterprise

**DOI:** 10.3389/fpsyg.2022.957531

**Published:** 2022-11-29

**Authors:** Ya-Wei Zhang, Wasi Ul Hassan Shah, Yu-Chun Xiao, Zahid Shafait

**Affiliations:** ^1^School of Management, Zhejiang Shuren University, Hangzhou, China; ^2^School of Management, Zhejiang Gongshang University, Hangzhou, China; ^3^College of Teacher Education, Zhejiang Normal University, Hangzhou, China

**Keywords:** multiteam systems, coordination patterns, case study, China, pharmaceutical enterprises

## Abstract

Effective coordination of multiteam systems (MTSs) can help enterprises respond quickly to complex and uncertain problems under disasters. However, it is unclear how MTS coordination patterns dynamically affect MTS performance in disasters. This study examined how MTSs responded to an emergency production incident at the Zhejiang Huisong Pharmaceutical Company in China during the COVID-19 pandemic through a qualitative and quantitative study. Based on social network theory, we found that a centralized coordination pattern impacts MTS performance by giving play to the leadership team’s network centrality position advantage during the crisis outbreak period. In the post-crisis period, the decentralized coordination pattern impacts MTS performance by giving play to the advantages of horizontal coordination. Our results help managers to consider the dynamics of coordination patterns in crisis management in ways that assist them in adapting an effective coordination pattern to changing and uncertain operational conditions.

## Introduction

Emergent crisis events are escalating globally. Over the years, crises such as Hurricanes Katrina and Sandy, the COVID-19 pandemic, and the recent Tonga volcanic eruption and ensuing tsunami have caused several million deaths and billions in damage. The scale of emergency crisis management, involving hundreds of staff and extensive technical requirements, is too large to manage as a single “team.” Two or more interdependent teams (departments) work to achieve team and system goals, as multiteam systems (MTSs) can respond effectively to such crises (Luciano et al., 2021). Such as the crash required MTS enforcement, i.e., medical teams, police, gendarmerie, firefighters, and emergency dispatch. Emergency rescue requires close collaboration between the local government and the hospital’s fire teams, on-site emergency medical teams, surgical teams, and rehabilitation teams to save the lives of the injured (Mathieu et al., 2001).

In reality, however, MTSs success is not easy. Luciano et al. (2018) proposed that the differentiation and dynamics of MTS would increase system uncertainty and undermine stability. The researchers point out that coordination is a crucial compensation process for stabilizing MTS (DeChurch and Marks, 2006). However, coordination is complex. Historically, coordination failures have been associated with slow responses (e.g., Hurricane Katrina; Leonard and Howitt, 2006). Dynamic changes in coordination patterns can quickly respond to environmental changes (e.g., military-civilian evacuation efforts following 9/11). Coordination patterns are the mode of action and interaction for information and exchange (Dietrich et al., 2013).

The two most common coordination patterns applied to MTSs are centralized and decentralized coordination. Information and knowledge exchange across component teams is centralized through the leadership team meeting, the primary inter-team coordination channel. Direct communication between component teams *via* Internet media, telephone, and face-to-face is part of decentralized coordination. Both coordination patterns have positively affected empirical studies (Dietrich et al., 2013). Existing studies explicitly define coordination patterns as an independent dimension and analyze them from a static perspective. We believe that defining the coordination patterns as a continuous dimension can more accurately understand the dynamics of MTS coordination patterns. It is unclear whether and how coordination patterns (centralized and decentralized coordinations) affect over time. In other words, the present study does not reveal how MTSs coordinate dynamically. Scholars have proposed that MTSs work in a complex and dynamic environment and the system needs to solve many problems, many of which may change over time (Mathieu et al., 2001; Zaccaro and DeChurch, 2012). Implementing dynamic adjustment of coordination patterns may provide timeliness in response to changing problems. However, how centralized and decentralized coordination patterns dynamically affect MTS performance during the crisis is still unknown. Understanding this process is vital because it helps explain why some MTSs respond quickly and share information, while others are slow. Our core research question: How do centralized and decentralized coordination patterns dynamically affect MTS performance in response to emergence?

Although scholars have paid attention to dynamics, the existing MTS coordination literature focused on the relatively rigid MTS (Davison et al., 2012; Bienefeld and Grote, 2013; Mell et al., 2020). They largely ignored the fluid nature of the MTS social network in response emergence. Division of labor theory and research show that a focused team will be functionally interdependent on the system’s work. That has a more significant influence on collective performance relative to other teams because it occupies a position more central to the workflow and performs special functions that are more important (Davison et al., 2012). It is essential to explore the relationship between coordination patterns and network centrality changes from the perspective of social networks, as such dynamics may lead to important adaptations. During the crisis outbreak period, emergency response MTSs can take advantage of the central position of the leadership team network through a centralized coordination pattern, reduce information redundancy and information overload, and quickly respond to environmental changes. During the post-crisis period, emergency response MTSs can take advantage of horizontal coordination (where component teams at the same level align and synchronize their activities). Through a decentralized coordination pattern, component teams can gain critical support from each other through horizontal coordination.

We investigated the emergency production activities of a pharmaceutical manufacturing company in Zhejiang, China, during the COVID-19 crisis, which was divided into three phases. Based on social network theory, centrality changes are correlated with different coordination patterns and MTS performance in three stages. A longitudinal case study and social network analysis (SNA) answered the above questions.

This study contributes to the literature and theory of MTSs and coordination in three critical ways. First, we theoretically argue the coordination patterns using qualitative and quantitative analysis of the MTS dynamic performance, in particular, the crisis outbreak period, the centralized coordination pattern to promote the MTS performance, the post-crisis period, and the distributed coordination pattern to promote the MTS performance, thus establishing a vital time theory for inter-team coordination and process. Second, we identify different mechanisms of the impact of centralized and decentralized coordination patterns on MTS performance in emergency response. Third, we tracked and investigated emergency response MTS in real extreme environments, providing a valuable methodological experience for testing MTS in real extreme environments.

## Theoretical background

### Centralized coordination pattern and multiteam systems performance

According to social network theory, MTSs can be conceptualized as a team network that generates different relationship patterns through interaction during task completion (Poole and Contractor, 2011). MTS is a part of a more complex dynamic network ecosystem. Luciano et al. (2018) believe that the structure of MTS has variability and instability over time, that is, the system structure configuration has dynamic centrality. Over time, network nodes are not static, and their roles or centrality may change significantly due to the environment (Braha and Bar-Yam, 2006). A focal component team may be central to achieving system goals at one point but less so at another (Davison et al., 2012). In the early stage of emergency response, the leadership team generally occupies the position of network centrality, which has an important impact on the rapid response of MTS in information sharing (Fodor and Flestea, 2016). Information sharing is the core of inter-team coordination (Marks et al., 2001; Mell et al., 2020). In healthcare settings, gaps in information sharing between healthcare teams during patient handovers have been shown to lead to adverse clinical outcomes for patients (Horwitz et al., 2008). In the centralized coordination pattern, information sharing and knowledge exchange are completed by leading team meetings among teams, and there is no direct information sharing among component teams. Davison et al. (2012) found that in MTSs, direct mutual adjustment among lower-level members of component teams is detrimental to performance and will only transform the MTS into an overly large, undifferentiated, and unwieldy team. Therefore, we believe that in the outbreak of a crisis, a centralized coordination pattern can play the advantage of leadership team centrality, reduce uncertainty, and improve the efficiency of decision-making and action. We propose Proposition 1:

In the crisis outbreak period, the centralized coordination pattern promotes the performance of MTS by giving play to the centrality position advantage of the leadership team.

### Decentralized coordination pattern and multiteam systems performance

Mathieu et al. (2001) believe that MTSs can be centralized or decentralized. The theory of MTSs does not formally dictate whether centralization or decentralization is most appropriate in a normative sense. To construct MTS dynamic theory, it is essential to understand when and how the decentralized coordination pattern affects MTS performance. Although Davison et al. (2012) believe that coordination activities among low-level component teams—horizontal coordination is not conducive to MTS performance. However, De Vries et al. (2016) found that high intrapersonal functional diversity could promote horizontal coordination and, thus, improve MTS performance. At the same time, Lanaj et al. (2013) suggested that the decentralization plan has both beneficial and harmful effects on MTS performance. Based on the social network theory, we believe that in the post-crisis period, environmental uncertainty and risk are reduced, leadership centrality is decreased, and the decentralization of decision-making power promotes the increase of horizontal coordination. The decentralized coordination pattern, in which the component teams directly share information face to face, can play a positive role in horizontal coordination and promote MTS performance by preventing redundancy and inconsistency between the activities of different component teams. We propose Proposition 2:

In the post-crisis period, the decentralized coordination pattern promotes MTS performance by giving play to the advantages of horizontal coordination.

## Materials and methods

### Case selection

Research is needed to examine MTSs in extreme environments to develop temporal theories of inter-team processes and understand how coordination may be improved within these challenging contexts (Waring, 2019). Investigating our research problems required the examination of an MTS to be tracked in a crisis. We studied the emergency production management of the Zhejiang Huisong Pharmaceutical Co., Ltd., China (Huisong hereafter), during the peak COVID-19 period from February to March 2020. Referring to the theoretical sampling method of Luciano et al. (2021), we believe that the above case is suitable for our study because (a) The MTS response to emergency production tasks during the COVID-19 is extensive, consisting of several teams working toward a common superior goal (i.e., production safety), but with unique sub-goals at the individual and team levels; (b) MTS operating in a crisis (during the severity of the COVID-19 pandemic); and (c) The structure of MTS is liquid at different times of crisis.

### Data collection

Data collection was divided into three stages. First, in February 2020, the researchers collected information on the resumption of work and production at the Huisong enterprise early in the epidemic. Second, in March 2020, after the outbreak of COVID-19 in Hangzhou, researchers tracked the work safety and production of the Huisong enterprise. Third, in April 2020, after there were no COVID-19 patients in Hangzhou, researchers followed up on the work safety situation of the Huisong enterprise.

The MTS comprised 23 men and 9 women. The average age was 33.48 years, and all were Chinese people. In MTS, there are 10 boundary or department managers, with an average age of 35 years, all with undergraduate education levels. There is one general manager, 48 years old, male, and with a high school education. We seek information sets among different data sources to improve the internal reliability of research results and help researchers have a deeper understanding of the same phenomenon (Jick, 1979). Primary data included semi-structured interviews and participatory observation data, and secondary data included pandemic publicity materials, meeting reports, news reports, official website updates, and social media publicity materials.

We designed a predefined interview framework according to Dietrich et al.’s (2013) definition of inter-team coordination patterns and interview design. The high quality of the interview frame and questionnaire was assured through discussions with experienced research colleagues before the start of the interview sessions. During the interview, in addition to asking the participants to explain the MTS structure, tasks, and key events in different periods of crisis, according to the design of Dietrich et al. (2013), the participants were also asked to determine the mechanism of information and knowledge exchange between teams in different periods. We explained to the participants the importance of the mechanism in facilitating the exchange of information and enhancing mutual understanding. Table 1 shows the semi-structured interview information.

**TABLE 1:** Case enterprise semi-structured interview information.

Position	Interview	Numbers	Duration
General manager	Composition and main events of the MTS in each stage	1	480 min
Boundary or department manager	Behavior and performance of each unit team at each stage	7	1,080 min
Other MTS members or department staff	Problems and solutions encountered in production safety	8	1,440 min

MTS, multiteam system.

### Data analysis

We used triangulation in the data analysis process to reduce response bias and enhance the validity of our findings (Johnson, 1997). We look for the integration of information from different data sources. Each key event has primary and secondary data sources to ensure that these findings are not just methodological artifacts.

**FIGURE 1:**
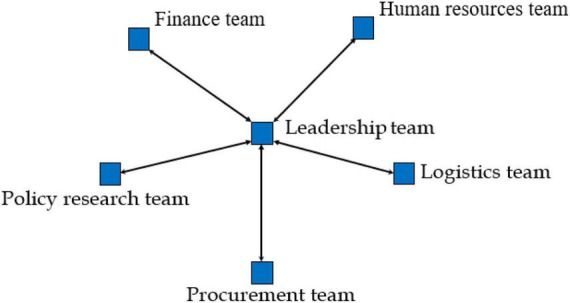
Multiteam system network structure in the crisis formation stage.

Our analysis strategy is as follows: (1) We organized all the text content according to the three-time frames corresponding to the emergency production task of the Huisong Enterprise that had emerged following preliminary data analysis: Stage 1, Crisis Formation (from 20 January to 9 February 2020; Figure 1); Stage 2, Crisis disruption (from 10 February to 28 February 2020, Figure 2); and Stage 3, Post-crisis (from 1 March to 30 March 2020, Figure 3), by analyzing all data sources and identifying the component teams involved (i.e., nodes) and their coordination (i.e., arcs—build coordination segments between network nodes for each episode, along with arrows—to indicate the direction of coordination). The analysis highlights the pattern of coordination between teams and dynamics during emergency production missions. We use the UCINET software to construct and analyze the MTS network. We used out-degree and in-degree to reflect component team centrality within the network. Out-degree centrality refers to the number of out-links of the component team, reflecting how easily the component team outputs relevant information in the network. In-degree centrality refers to the number of in-links of the component team, reflecting the ease with which component teams can receive relevant information in the network.

**FIGURE 2:**
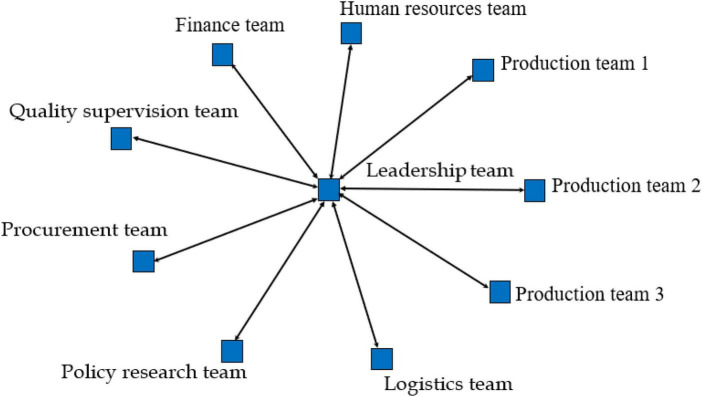
Multiteam system network structure in the crisis destruction stage.

**FIGURE 3:**
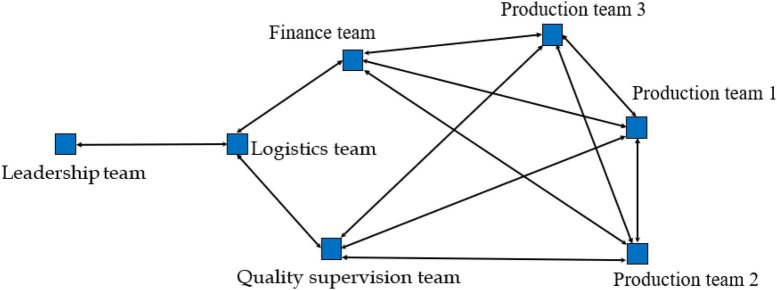
Multiteam system network structure in the post-crisis stage.

(2) In addition to using network analysis to explore the coordination patterns, we also used key crisis events analysis on the raw data to reflect on the influence of the MTS coordination pattern over the MTS performance.

## Case description

In early 2020, 44 COVID-19 cases were detected in Wuhan, Hubei, China, spreading countrywide. Pharmaceutical companies need to risk production and expand capacity to control the spread of the virus. Huisong resumed production on 2nd February and established emergency production MTS to manage production tasks on 7th February by the municipal government policy. The MTS encountered problems such as unclear communication of safety information, workers’ inability to arrive at their posts, and inability to restore production capacity. After more than 2 months, by 15 March 2020, Huisong had no infections, and its capacity had returned to normal; the production at Huisong exceeded that of local enterprises by 80%.

This study divides Huisong’s work safety management incidents into three stages—crisis formation, crisis destruction, and post-crisis—based on the risk and uncertainty of COVID-19 perceived by MTS members at different stages. In the crisis formation stage, COVID-19 spread rapidly, and enterprises needed to ensure personnel safety and plan production tasks. MTS had to formulate production safety plans and systems according to government policies. In the crisis destruction stage, the pandemic situation changed rapidly. Huisong experienced a lack of both personnel and materials. In the post-crisis stage, the pandemic was effectively controlled. Huisong had no infected personnel, and its productivity increased.

### Crisis formation stage: Proactive planning

This MTS stage involves the leadership, policy research, procurement, human resources, finance, and logistics teams. On 7 February 2020, general manager A identified himself and other team leaders as the leadership team for work safety management during the pandemic; other component teams comprised two to three people from different departments. The main work of MTS in this stage was preparing pandemic prevention materials according to the changeable policies and establishing a safe production system. MTS’s goal was to determine component teams’ safety on duty and apply to the government to resume production. Key events A1 to A3 are extracted from this stage (refer to Table 2).

**TABLE 2:** Main events of Multiteam system (MTS) leadership, coordination, and performance in the crisis formation stage.

Event	Main codes	Case description
A1	Centralized coordination pattern	The policy research team will study relevant resumption policies according to the requirements of the leadership team and report them to the leadership team. (Policy research team member, interview, meeting book)
A2	Centralized coordination pattern	The leadership team conference analyzed Huisong’s internal and external difficulties and prepared application materials to resume work. (Meeting book) “During special periods, everything should be reported to the leadership team and distributed at the leadership team meeting” (General manager, interview)
A3	MTS performance	A total of 39 people in the enterprise met the requirements of the government to resume work, and the government approved 39 people to resume work at 16:00 on February 9. (Meeting book, Huisong’s official website)

MTSs reduced the spread of unclear or invalid information through a centralized coordination pattern, strengthened enterprises’ monitoring, and simplified cooperation. The centralized coordination pattern thus ensures MTS performance in the crisis formation stage.

### Crisis destruction stage: Control action

In this stage, pandemic-related risks and uncertainties intensified further. There were 10 COVID-19 cases in Dingqiao, Hangzhou. Communities in Dingqiao closed down, production workers were reduced, production workshop capacity was reduced, and conflicts between component teams intensified. While Huisong needed to resume its production scale actively, it also had to abide by the government’s pandemic prevention policies to ensure personnel safety and avoid the enterprise’s isolation. Specifically, the leadership team continued adopting top-down interactive conflict management and strengthening centralized coordination patterns. MTS in this stage involves the leadership, procurement, human resources, quality supervision, production, finance, and logistics teams. MTS targets in this stage include production and safety, involving key events B1 to B3 (refer to Table 3).

**TABLE 3:** Main events of multiteam system (MTS) leadership, coordination, and performance in the crisis destruction stage.

Event	Main codes	Case description
B1	Centralized coordination pattern	Some production workers were reluctant to come to work for fear of infection, and some who were willing to come did not have a clear itinerary. There was a dispute within MTS about how to deal with these employees. The leadership team held a special meeting to discuss this. If the human resources team reported that the sixth worker might be at risk, it was necessary to report and track the movement and location of the person. (Human resources team manager, interview, meeting book)
B2	Centralized coordination pattern	On February 12, Dingqiao area is closed due to COVID-19 in HangZhou; people living in Dingqiao will not come to work. The H.R. team learned that four employees had been isolated from Dingqiao and reported this to the leadership team. The leadership team meeting determines the reduction of production and asks the production team to find additional workers as soon as possible. (Leadership team, interview, meeting book)
B3	MTS performance	Safety precautions are in place and no human infection has occurred. There is a restoration of 30% capacity. (General manager, interview)

### Post-crisis stage: Action adjustment

In March, the number of COVID-19 infections in China decreased significantly. Most employees returned to the factory, and MTS needed to ensure infection prevention and restore production capacity as soon as possible. MTS involves the leadership, quality supervision, production, finance, and logistics teams. The leadership team meeting was weakened, and spontaneous interaction between component teams constituted a decentralized coordination pattern to achieve production and safety performance goals. It involves key events C1 and C2 (refer to Table 4).

**TABLE 4:** Main events of multiteam system (MTS) leadership, coordination, and performance in the post-crisis stage.

Event	Main codes	Case description
C1	Decentralized coordination pattern	There will be direct coordination between the production team, the quality team, and the logistics team. If the quality team finds any problem, it will directly contact them (the production team manager), and they will check the problem. (Quality team manager, interview) “When normalcy is restored, our team (production team) does not have to consult the leadership team.” (Production team manager, interview)
C2	MTS performance	The company resumes normal production, and no human infection is reported. There is the restoration of 80% capacity. (Observation and interview)

## Results

From the coding of coordination patterns, in Stages 1 and 2, MTS ultimately adopts a centralized coordination pattern to complete information exchange and knowledge flow, that is, leadership team meetings. SNA further validates this result. Table 5 shows that the in-degree and out-degree centralities of the leadership team in stages 1 and 2 are one standard deviation above the mean, which means that the leadership team is at the center of the network. During the outbreak of the crisis, the centralized coordination pattern can take advantage of the leadership team’s position in the center of the network, and quickly filter the invalid information in the input and output of information. Avoid situations in which important details are overlooked between teams, which could lead to a delay in response or an ineffective course of action. According to the company’s operating data given by the MTS general manager, Huisong achieved the production safety requirements required by the government in Stages 1 and 2. The results verify Proposition 1: centralized coordination pattern can play the advantage of leadership team network centrality position and achieve MTS performance in a crisis outbreak period.

**TABLE 5:** Node out-degree centrality and in-degree centrality in three performance stages of the multiteam system.

	Stage 1: Proactive planning		Stage 2: Control action		Stage 3: Action adjustment	
	Out-degree	In-degree	Out-degree	In-degree	Out-degree	In-degree
Leadership team	5	5	7	7	1	1
Policy research team	1	1	–	–	–	–
Human resources team	1	1	1	1	–	–
Procurement team	1	1	1	1	–	–
Finance team	1	1	1	1	4	4
Logistics team	1	1	1	1	3	3
Production team 1	–	–	1	1	4	4
Production team 2	–	–	1	1	4	4
Production team 3	–	–	1	1	4	4
Quality supervision team	–	–	–	–	4	4
Mean (m)	1.67	1.67	1.75	1.75	3.43	3.43
Standard deviation (SD)	1.49	1.49	1.98	1.98	1.05	1.05
m + SD	3.16	3.16	3.73	3.73	4.48	4.48
Network centrality	80%	80%	85.71%	85.71%	11.11%	11.11%

It extends previous research stating that leaders with high network centrality are beneficial in crises. For instance, leaders at the network’s center can filter errors and invalid information, communicate correct information to component teams that need it, and reduce conflict between component teams, thus positively affecting system-level coordination and performance (Balkundi and Harrison, 2006).

From the perspective of the coding of the coordination pattern, in stage 3, MTS ultimately adopts the decentralized coordination pattern to complete information exchange and knowledge flow; that is, the component teams cooperate directly through face-to-face or WeChat. The SNA further verifies this result. Table 5 shows that the leadership team’s centrality of out-degree and in-degree in stage 3 is lower than the mean value by one standard deviation, which means that the leadership team is no longer a collaboration center. In the post-crisis period, the decentralized coordination pattern can take advantage of direct coordination among component teams (i.e., horizontal coordination) and promote the performance of MTSs by preventing redundancy and inconsistency between activities of different component teams (Hoegl et al., 2004; Lanaj et al., 2013). In addition, component teams can gain critical support from each other through horizontal coordination, which enables them to perform better the critical tasks of the system as a whole (Joshi et al., 2009). According to the company’s operating data given by the MTS general manager, the production of Huisong recovered to the pre-pandemic level in phase 3. The results verify Proposition 2: In the post-crisis period, decentralized coordination mode can play the advantages of horizontal coordination and promote MTS performance.

## Discussion

We examined emergency response MTS during COVID-19 in Chinese pharmaceutical companies and answered the research question: How do MTS coordination patterns dynamically affect MTS performance in a crisis? From the perspective of social networks, we find that a centralized coordination pattern achieves MTS performance by giving play to the leadership team’s network centrality position advantage during the crisis outbreak period. In the post-crisis period, the decentralized coordination pattern promotes MTS performance by giving play to the advantages of horizontal coordination. Taking these findings together, we propose an empirically based, performance episode continuous, and social network theory-based dynamic model of MTS coordination patterns. This reveals the continuity of different MTS coordination patterns and their dynamic impact on MTS performance.

### Theoretical implications

Our study establishes a new theory of how MTS coordination patterns dynamically impact MTS performance. Notably, insights from social network theory regarding network centrality make it possible to identify differential processes by coordination patterns that dynamically affect MTS’s effectiveness. More specifically, our findings shed light on how MTS coordination patterns benefit MTS in different performance episodes (the crisis outbreak period and the post-crisis period). We show the importance of adopting a centralized coordination pattern during the crisis outbreak period to make rapid decisions and respond to environmental changes. Understanding the impact of centralized and decentralized coordination patterns on MTS performance in different performance episodes shifts the focus from overall MTS performance to process performance.

Focusing on the influence of coordination patterns dynamically impacting MTS performance from the perspective of social networks can not only provide a tool kit but also provides more accurate and testable suggestions for the operation of inter-team interdependence and dynamic interaction, which is conducive to developing a more dynamic theory of MTS. The conclusion that the centralized coordination (decentralized coordination pattern) pattern can play the advantage of the central position of the leadership team network (horizontal coordination) in the crisis outbreak period (post-crisis period) is echoed by Mathieu et al. (2019) who states that the concept of team research is dynamic network. This conclusion also has important implications for constructs theorized to have dynamic properties of MTS but have not yet been tested by empirical studies (e.g., MTS Effectiveness, Turner et al., 2020). By examining the dynamic effects whenever and wherever they arise, the MTS literature may develop a more nuanced and actionable understanding of the dynamic effects of MTS.

Our findings also advance the understanding of horizontal coordination in MTS. Davison et al. (2012) found that in MTSs, direct mutual adjustment among component teams (i.e., horizontal coordination) is detrimental to performance and will only transform the MTS into an overly large, undifferentiated, and unwieldy team. The literature on small independent teams primarily considers positive decentralized practices, while theoretical work on large organizations shows that these practices have positive and negative characteristics (Lanaj et al., 2013). As for the external features of MTS research (such as decentralization planning), (Lanaj et al., 2013) and intrapersonal functional diversity will affect horizontal coordination (De Vries et al., 2016). Our findings further expand our understanding of the dynamic effects of horizontal coordination, showing where and when horizontal coordination can have a positive effect. More specifically, they treat horizontal coordination as a static variable and consider the impact of external interventions on horizontal coordination, which is a simplification that ignores the potential positivity of coordination patterns over time. Therefore, scholars should take a more dynamic and multifaceted approach to the coordination mechanism and pay careful attention to its connection with the dynamic attributes of MTS.

### Managerial implications

Regarding practical implications, first, to deal with crises, enterprises should rely on management and control in the conventional situation to prevent risks and adopt different coordination patterns based on changes in the crisis. The original team’s process design should be changed during coordination, and a rapid approval and response mechanism should be developed. Second, leaders should quickly adjust their cognition according to changes in the crisis and emphasize differentiated management of the leadership team at different emergency response stages. It strengthens unified management in the early and middle crisis stages, reduces the spread of unclear or invalid information, strengthens empowerment and incentives in the post-crisis period, and maximizes capacity recovery.

### Limitations

Although this study obtains valuable results on the dynamic of MTS coordination in Chinese enterprises during crises, some deficiencies need to be addressed in future research. The insights derived from our research are likely to be most easily adapted to the emergency production environment of a manufacturing enterprise. However, other organizations may also encounter dynamic complexity challenges that require rapid response, such as an executive sex scandal or severe product quality issues that require rapid response and coordination among several teams within the organization. We hope that future studies will explore our theory’s boundary conditions by examining other organizations’ emergency response activities. Although Huisong has achieved phased victory during the pandemic, this crisis is not entirely over. In the future, continuous attention should be paid to its development and changes to enrich existing research further. Future studies can further investigate the impact of emergency states and processes on the effectiveness of MTS in dynamic environments by using social network methods.

## Conclusion

We collected data from the emergency production activities of pharmaceutical manufacturers during the COVID-19 pandemic. Based on social network theory, we found that a centralized coordination pattern impacts MTS performance by giving play to the leadership team’s network centrality position advantage during the crisis outbreak period. In the post-crisis period, the decentralized coordination pattern impacts MTS performance by giving play to the advantages of horizontal coordination. From the dynamic perspective, the study finds that the coordination patterns can resist the destructive force brought by the change of MTS structure under crisis.

## Data availability statement

The raw data supporting the conclusions of this article will be made available by the authors, without undue reservation.

## Ethics statement

The studies involving human participants were reviewed and approved by the Ethics Committee of Zhejiang Shuren University, Hangzhou, 310015, China. The patients/participants provided their written informed consent to participate in this study.

## Author contributions

All authors listed have made a substantial, direct, and intellectual contribution to the work, and approved it for publication.
